# Supplementary material for: An Insight into Occurrence, Biology, and Pathogenesis of Rice Root-Knot Nematode Meloidogyne graminicola
Source: Biology (Basel). 2023 Jul 11;12(7):987. doi: 10.3390/biology12070987 (PMC10376547; doi:10.3390/biology12070987)

Supplementary file 1.

I. **Testing of the Protein Models using Ramachandran Plot using the web server SAVES SERVER(**[https://saves.mbi.ucla.edu/)](https://saves.mbi.ucla.edu/)

1.Phospholipase B like Protein

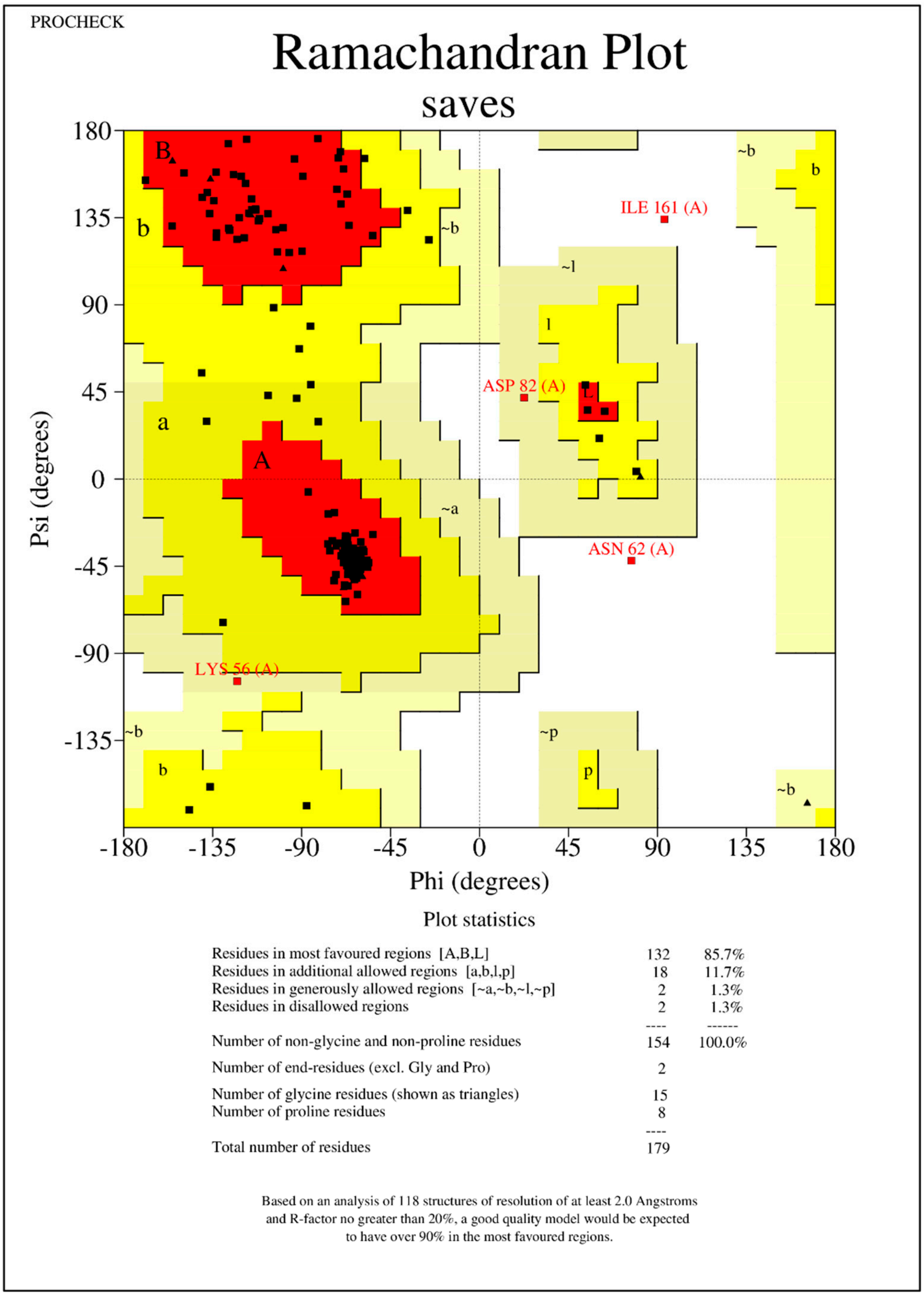

saves_01.ps

2. G protein couple receptor

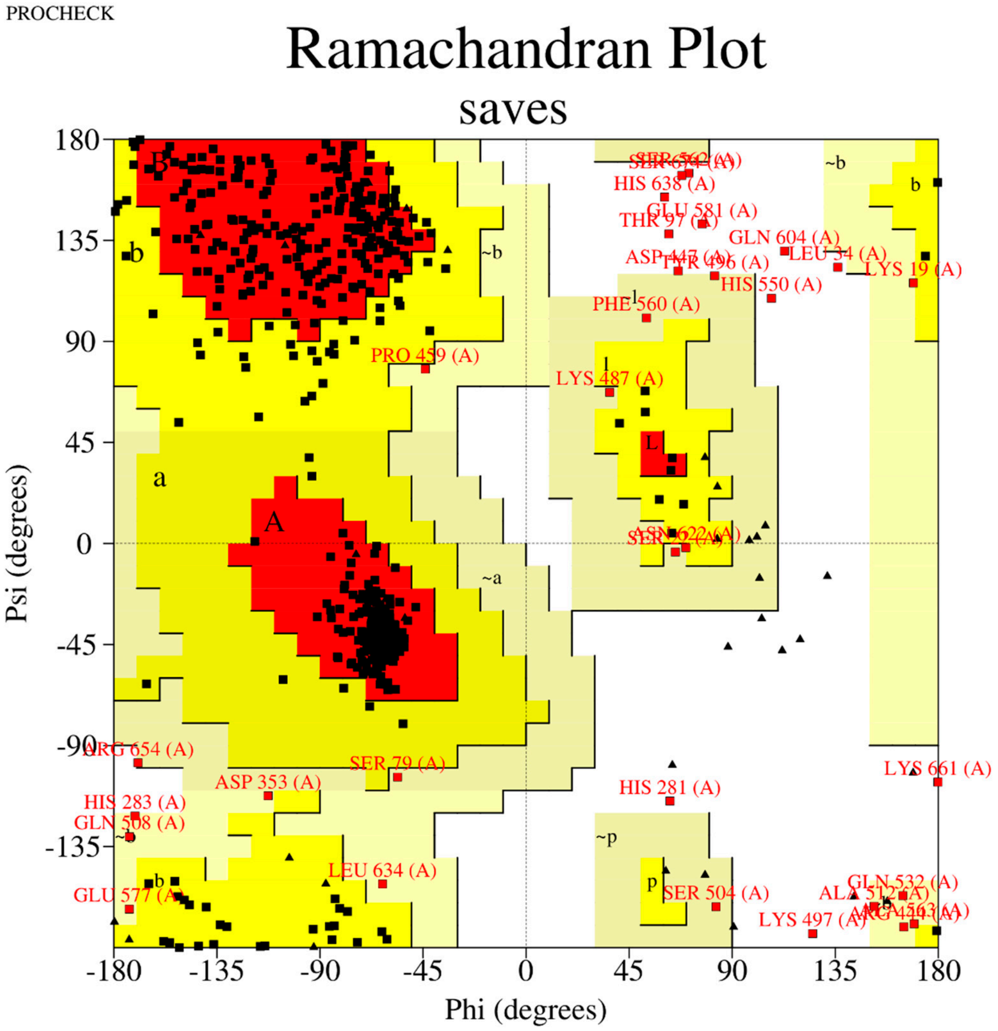

Plot statistics

Residues in most favoured regions [A,B,L]	512	81.9%
Residues in additional allowed regions [a,b,l,p]	83	13.3%
Residues in generously allowed regions [~a,~b,~l,~p]	18	2.9%
Residues in disallowed regions	12	1.9%
	----	------
Number of non-glycine and non-proline residues	625	100.0%
Number of end-residues (excl. Gly and Pro)	2	
Number of glycine residues (shown as triangles)	41	
Number of proline residues	27	

Total number of residues	695	

Based on an analysis of 118 structures of resolution of at least 2.0 Angstroms and R-factor no greater than 20%, a good quality model would be expected to have over 90% in the most favoured regions.

saves_01.ps

3. Endoglucanase

PROCHECK

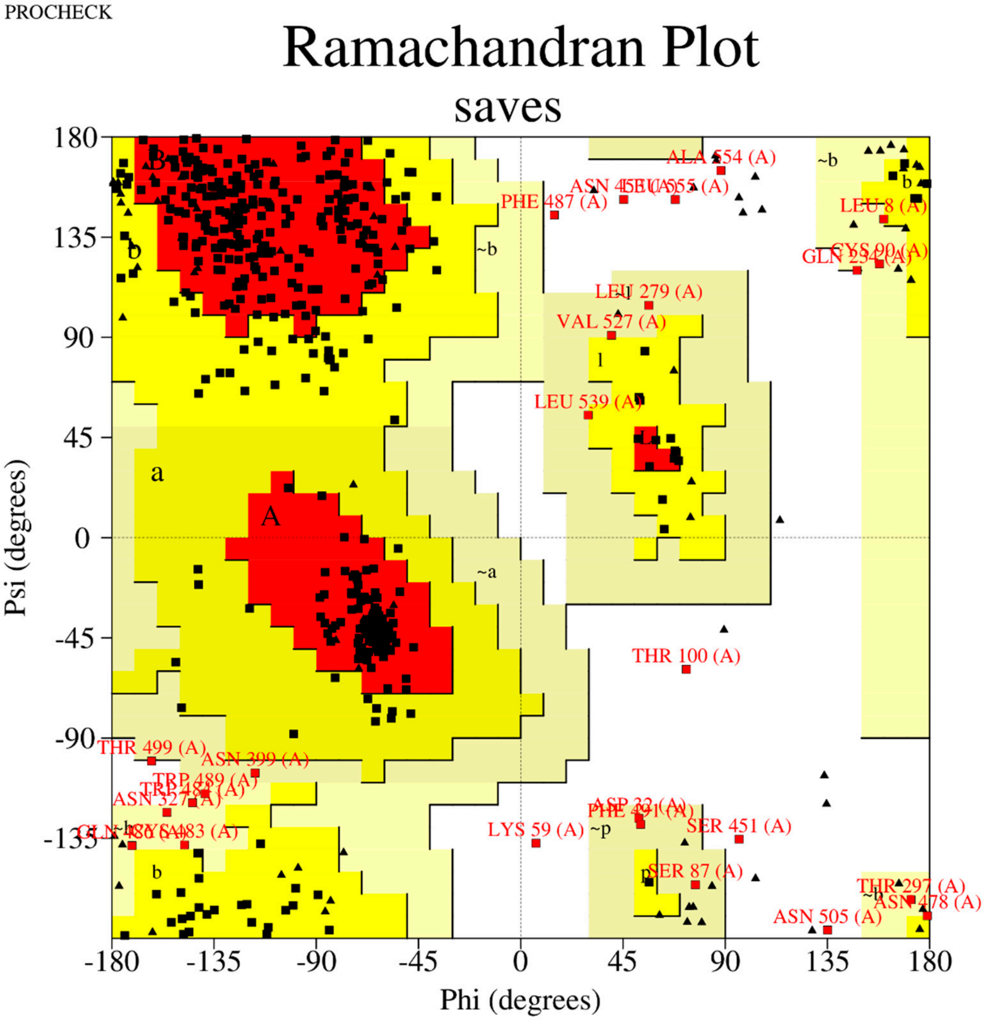

Plot statistics

Residues in most favoured regions [A,B,L]	363	75.2%
Residues in additional allowed regions [a,b,l,p]	94	19.5%
Residues in generously allowed regions [~a,~b,~l,~p]	17	3.5%
Residues in disallowed regions	9	1.9%
	----	------
Number of non-glycine and non-proline residues	483	100.0%
Number of end-residues (excl. Gly and Pro)	2	
Number of glycine residues (shown as triangles)	94	
Number of proline residues	15	

Total number of residues	594	

Based on an analysis of 118 structures of resolution of at least 2.0 Angstroms and R-factor no greater than 20%, a good quality model would be expected to have over 90% in the most favoured regions.

saves_01.ps

4. Pectate lyase

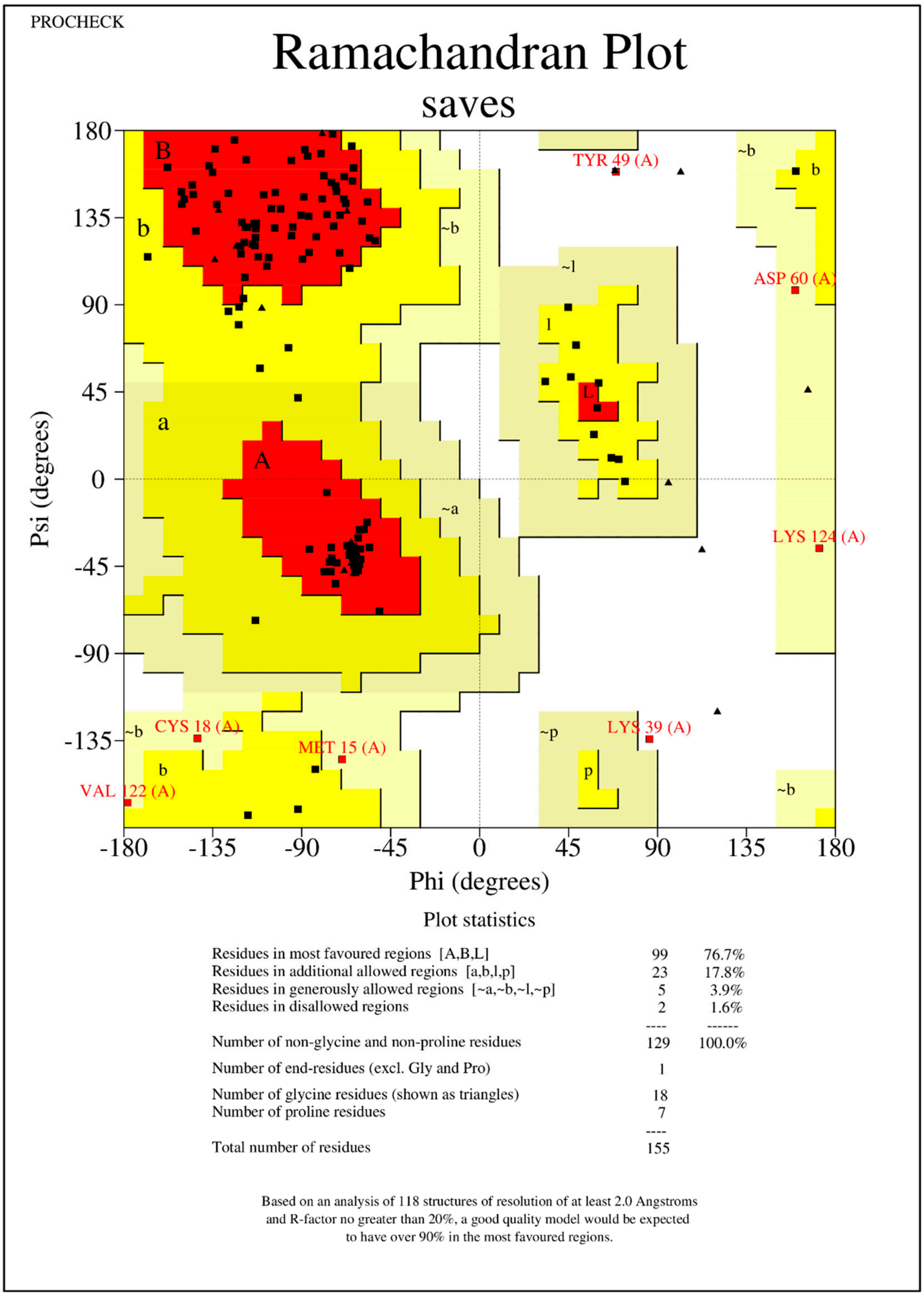

saves_01.ps

II. Developed protein models for molecular docking studies

a. **G Protein receptor kinase**

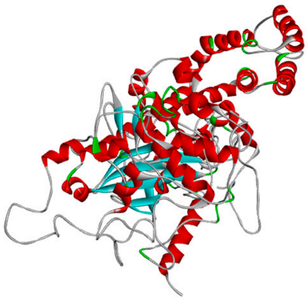

b. **Beta 1,4 endoglucanase**

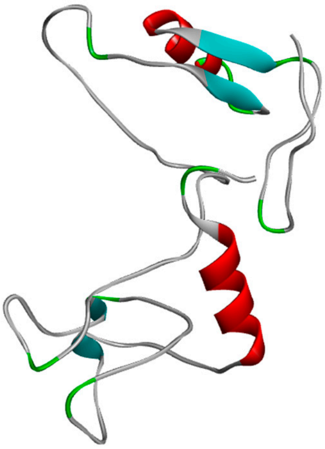

c. **Phospholipase**

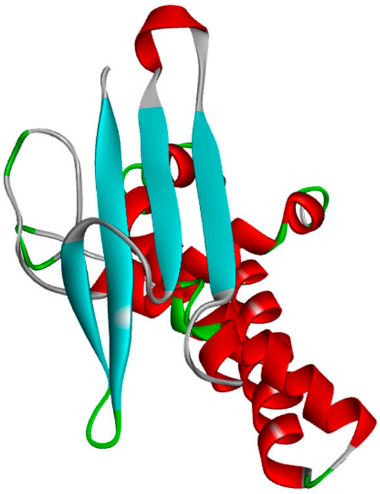

d. **Pectate lyase**

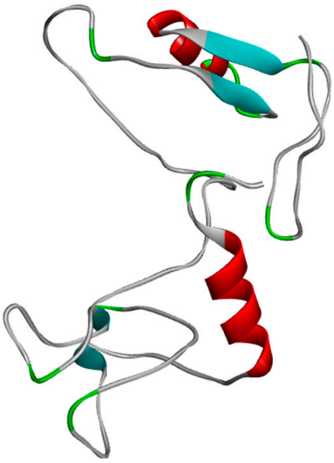

III. Binding affinity (kcal/mol) Of small molecules on different virulent proteins of *M. graminicola*

a. **Pectate lyase vs 4-O-Beta-D-Galactopyranosyl-Alpha-D-Glucopyranose**

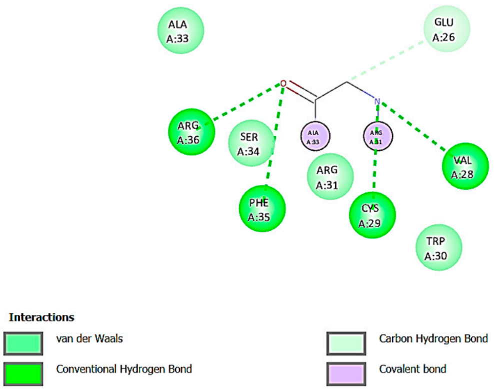

b. **Pectate lyase vs Beta-D-Galacturonic Acid**

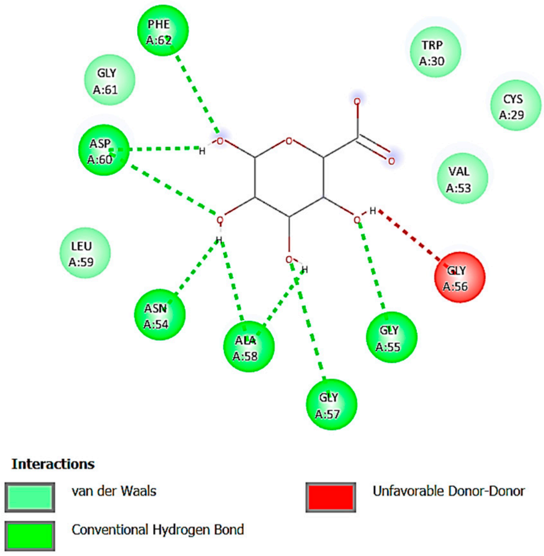

c. **Pectate lyase vs (2S)-2-amino-3-phenylpropanoic acid**

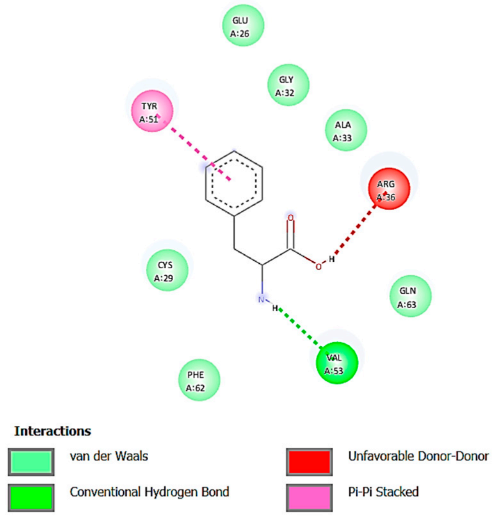

d. **Pectate lyase vs 2,6,10,15,19,23-hexamethyltetracosane**

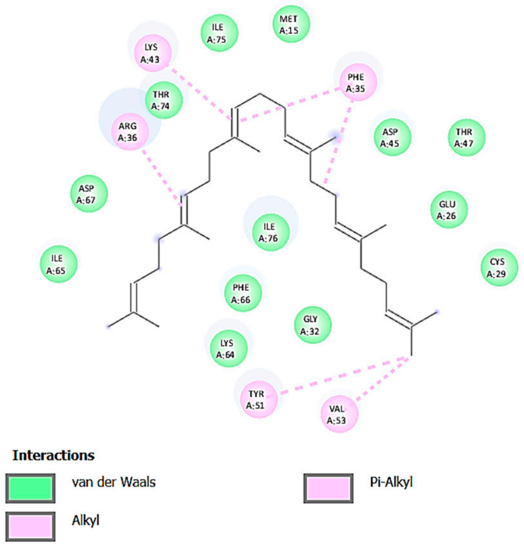

e. **G Protein kinase vs (2S)-2-amino-3-phenylpropanoic acid**

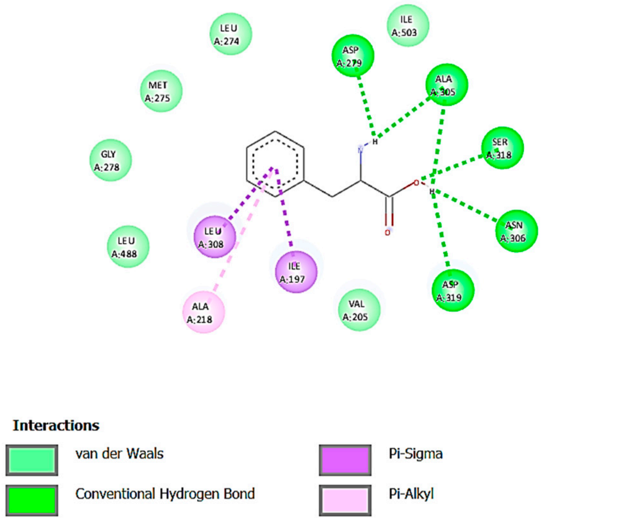

f. **G Protein kinase vs 4-O-Beta-D-Galactopyranosyl-Alpha-D-Glucopyranose**

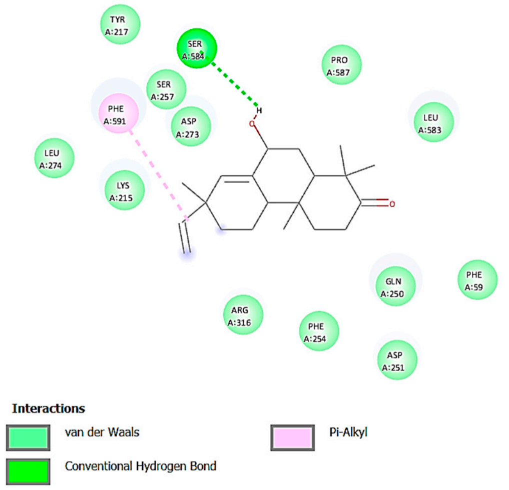

g. **G Protein kinase vs Beta-D-Galacturonic Acid**

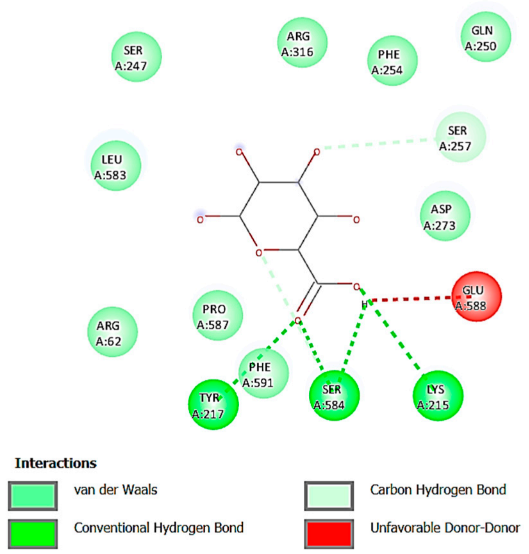

h. **G Protein kinase vs 2,6,10,15,19,23-hexamethyltetracosane**

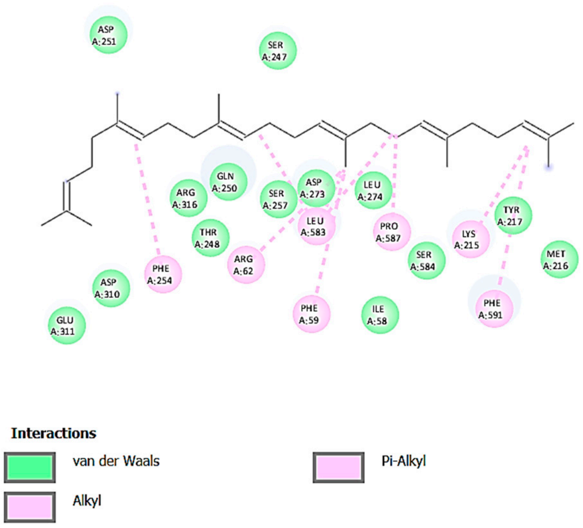

i. **b-1,4-endoglucanase vs 2,6,10,15,19,23-hexamethyltetracosane**

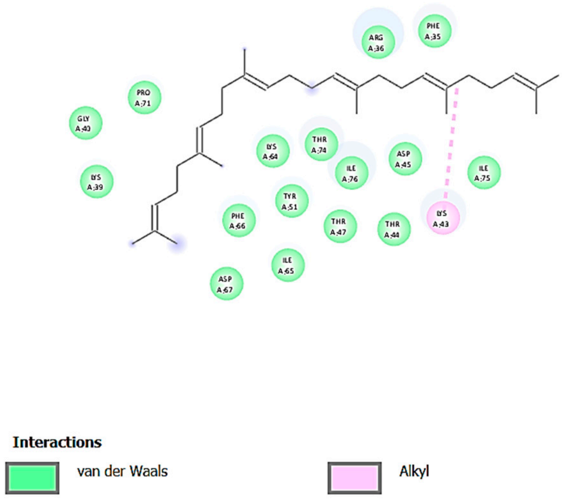

j. b-1,4-endoglucanase vs (2S)-2-amino-3-phenylpropanoic acid

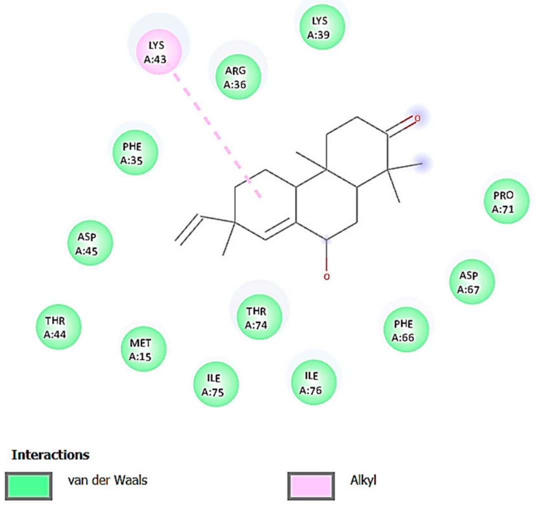

k. b-1,4-endoglucanase vs 4-O-Beta-D-Galactopyranosyl-Alpha-D-Glucopyranose

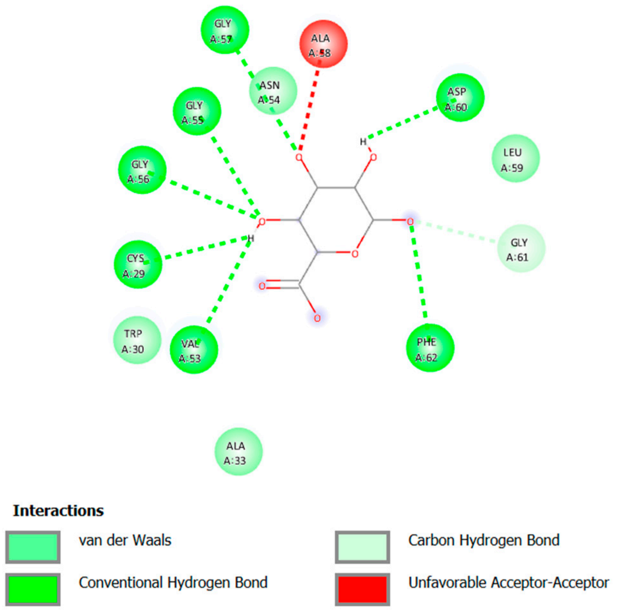

l. **b-1,4-endoglucanase vs Beta-D-Galacturonic Acid**

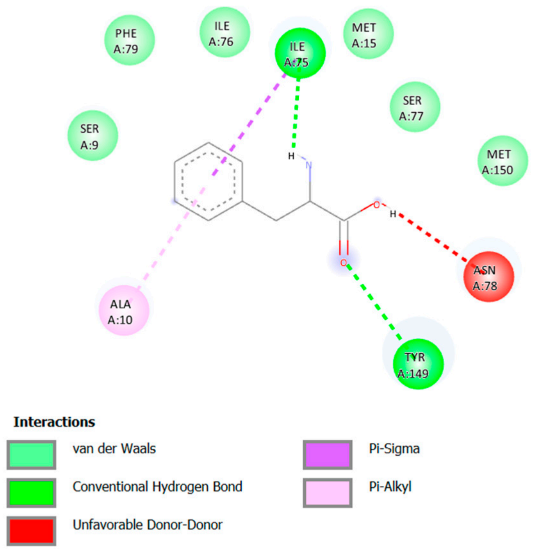

m. **Phospholipase vs Beta-D-Galacturonic Acid**

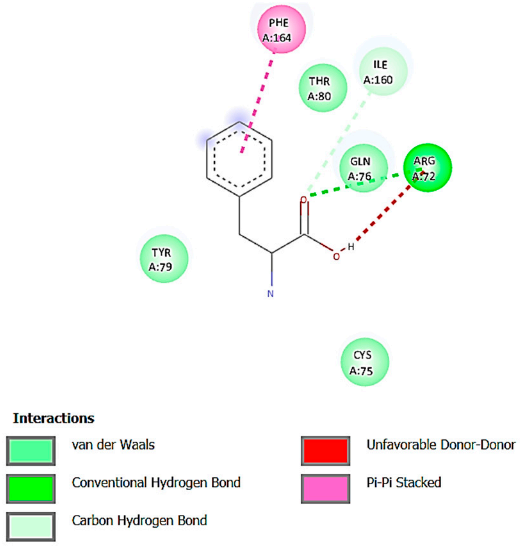

n. **Phospholipase vs (2S)-2-amino-3-phenylpropanoic acid**

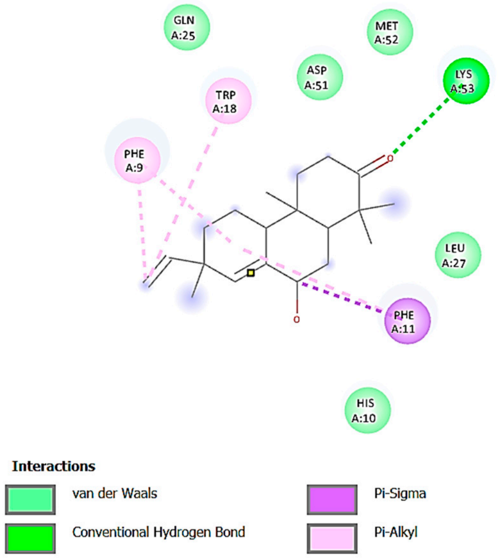

o. **Phospholipase vs 4-O-Beta-D-Galactopyranosyl-Alpha-D-Glucopyranose**

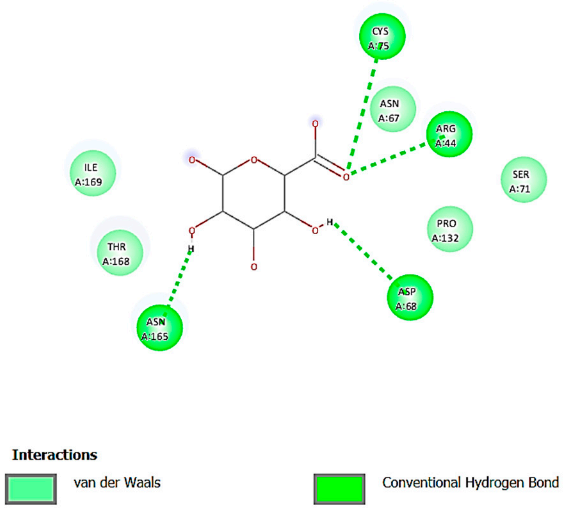

p. **Phospholipase vs 2,6,10,15,19,23-hexamethyltetracosane**

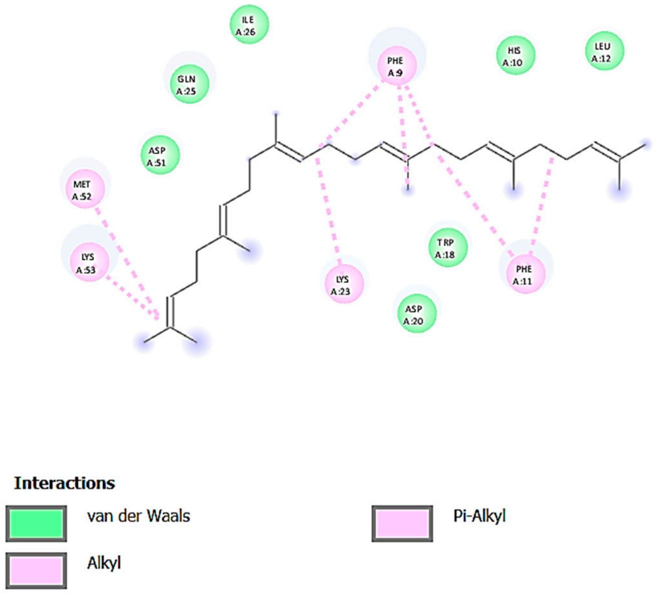

Supplement: Supplementary file 1 [file biology-12-00987-s001.zip › biology-2329294-supplementary.pdf]
